# Process evaluation of a university residence-based SARS-CoV-2 testing programme in the UK

**DOI:** 10.1093/eurpub/ckac131.046

**Published:** 2022-10-25

**Authors:** H Blake, S Carlisle, L Fothergill, J Hassard, A Favier, J Corner, JK Ball, C Denning

**Affiliations:** 1 School of Health Sciences, University of Nottingham, Nottingham, UK; 2 School of Medicine, University of Nottingham, Nottingham, UK; 3 Faculty of Registrars, University of Nottingham, Nottingham, UK; 4 Executive Office, University of Nottingham, Nottingham, UK; 5 School of Life Sciences, University of Nottingham, Nottingham, UK; 6 Biodiscovery Institute, University of Nottingham, Nottingham, UK; 7 NIHR Nottingham Biomedical Research Centre, University of Nottingham, Nottingham, UK

## Abstract

**Background:**

Regular testing for SARS-CoV-2 is an important strategy for controlling virus outbreaks on university campuses during the COVID-19 pandemic but testing participation can be low. The Residence-Based Testing Participation Pilot (RB-TPP) was a novel 4-week intervention implemented at two student residences on a UK university campus, aiming to increase asymptomatic testing frequency and normalise university life through relaxed social restrictions onsite.

**Methods:**

Mixed-methods process evaluation determined whether RB-TPP was implemented as planned and identified implementation barriers and facilitators. Data were collected from meeting records, university students (online survey: n = 152; focus groups: n = 30), and staff (interviews, n = 13). Barriers and facilitators to implementation were mapped to the ‘Capability, Opportunity, Motivation-Behaviour’ (COM-B) behaviour change framework.

**Results:**

Uptake was high (n = 464 students opted-in; 98% of those living onsite). Implementation was broadly as planned, with adjustments due to national escalation of the COVID-19 Delta variant. Majority engaged in testing (88%); 46% (52% of testers) were fully compliant with pre-determined testing frequency. Most felt positively towards relaxed social distancing (97.9%). Implementation was facilitated by convenience and efficiency of testing and reduced negative impacts of isolation through opportunities for students to socialise. Barriers to implementation were mixed-messages about the rules, ambivalent attitudes, and lack of adherence to COVID-19 protective measures in the minority.

**Conclusions:**

This is the first process evaluation of the implementation of asymptomatic SARS-CoV-2 testing in university residences. Testing participation increased and student mental wellbeing improved. Rapid adaptions to the changing pandemic context generated complexity and challenge. Findings have global relevance for outbreak prevention and management strategies in higher education settings.

**Key messages:**

• Delivery of asymptomatic SARS-CoV-2 testing and relaxation of social distancing within residences led to high rates of testing participation and benefits for student mental wellbeing.

• This is the first process evaluation of the implementation of asymptomatic SARS-CoV-2 testing in university residences with global relevance for outbreak prevention in higher education settings.

